# Nevus pigmentosus et pilosus

**DOI:** 10.11604/pamj.2021.40.107.26779

**Published:** 2021-10-18

**Authors:** Supraja Nagarathinam, Krishna Prasanth Baalann

**Affiliations:** 1 Department of Community Medicine, Sree Balaji Medical College and Hospital, Bharath Institute of Higher Education and Research, Chennai, Tamil Nadu, India

**Keywords:** Congenital melanocytic nevus, melanoma, dermabrasion

## Abstract

Congenital melanocytic nevus (CMN) is a proliferation of melanocytes that presents at birth or shortly after birth as light brown to black patches or plaques, covering any part of the body occasionally exhibiting hypertrichosis. The estimated prevalence of such large forms is 0.002% of the births, resulting from mutations of genes coding for NRAS and KRAS proteins, usually during the first twelve weeks of pregnancy. Giant CMN also known as “bathing trunk nevus,” “giant hairy nevus”, and “nevus pigmentosus et pilosus” has highest potential to turn into malignant melanoma. We present a case of a 7 year old boy who came to our clinic with complaints of itching over his left shoulder and back for 4 months duration. He gave history of black patches on those areas since birth that gradually grew in size and attained the current presentation. On examination, well demarcated black patches were noted over his left shoulder, arm, trunk (A) and extending from nape of the neck and the entire back with excessive hair growth (B). A diagnosis of congenital melanocytic nevus was established and since it could potentially evolve into a melanoma, prophylactic surgery with skin grafting was suggested but the patient's parents refused. Hence dermabrasion was done to lighten the dark pigmentation and reduce hair growth within nevi. Parents were counselled about complications of CMN and advised to follow up with a dermatologist regularly. Clinicians should scrupulously examine the child to facilitate timely surgical intervention thereby reducing morbidity and mortality associated with this condition.

**Figure 1:**
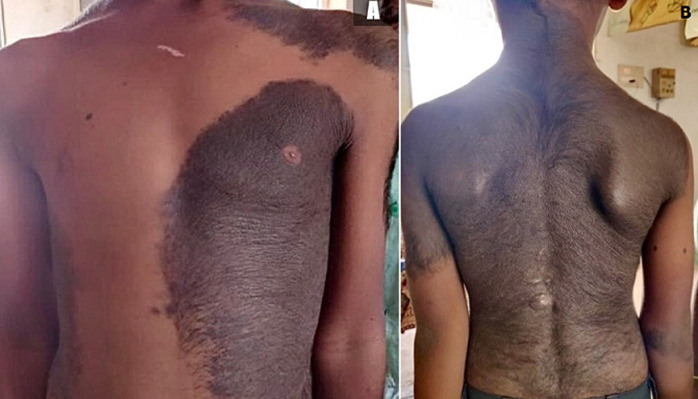
A) well demarcated black patches over left shoulder and trunk; B) black pigmentation extending from nape of neck to lower back with hypertrichosis

